# Miscellaneous

**Published:** 1889-11

**Authors:** 


### MISCELLANEOUS.

### PRACTICAL VEGETARIANS.

"Rev. Sylvester Graham and William Andrus Allcott, M. D., began the practice and advocacy of Vegetarianism about the same time, and in entire ignorance of each other. The latter published *Vegetable, Diet* in 1849, but gave up the use of flesh meat in 1830. He first committed his views to print in 1832.

"Sylvester Graham lived from 1794 to 1851. In 1830 he began lceturing on temperance, and his studies upon this subject subsequently led him to diet reform. His classic work, *The Science of Human Life*, was published in 1839, while his brilliant and profound advocacy of Vegetarianism originated the American words *Grahamism* and *Grahamite*, which are now disappearing, though the adjective *graham* still attaches to whole-wheat meal and flour.

"It will be seen, therefore, that any honor there may be in first adopting and advocating the system, belongs to Dr. Alcott.

"But, earlier still, Benjamin Franklin for a time, and many others here and there, had practiced the same reform.

"Amos Bronson Alcott, a cousin of Dr. Alcott, left off flesh-eating in 1835.

"Rev. William Metcalfe, M. D., was born in England in 1788, and at the age of twenty-one was converted to a new diet. He soon came to Philadelphia, where he preached until his death in 1862. Apparently we must count him the father of American Vegetarianism, as well as total abstinence."

Mrs. Sally B. Weeks Bucknam, then a blushing bride, went to housekeeping seventy-three years ago in a snug farmhouse on the west slope of Mount Prospect N. H. The other day in the same farmhouse, where she had lived ever since, she celebrated her one hundreth birthday, and was strong enough to receive not only her children, grandchildren and great grandchildren, but also a large number of her friends and acquaintances.

### GEESE SWALLOWED BY SNAKES.

A farmer on Bullskin Prarie, Ind., had a drove of twelve half grown geese killed and swallowed by rattle-snakes. The geese were observed early in the day by a gang of telephone men at work on the Salamonie line, and their strange actions were commented on, but the cause was not discovered until toward evening, when the one remaining goose was rescued from a circle of rattle-snakes and several of the reptiles were killed, their bellies distended with the geese they had swallowed. One of the rattlers was an enormous fellow about five feet in length.

### HURRIED DINNERS.

It is a mistake to eat quickly. Mastication performed in haste must be imperfect even with the best of teeth, and due admixture of salivary secretion with the food cannot take place. When a hugh mass of inadequately crushed muscular fibre, or undivided solid material of any description, is thrown into the stomach, it acts as a mechanical irritant, and sets up a condition in the mucous membrane lining of that organ which greatly impedes, if it does not altogether prevent, the process of digestion. When the practice of eating quickly and filling the stomach with unprepared food is habitual, the digestive organ is rendered incapable of performing its proper functions. Either a much larger quantity of food than would be necessary under natural conditions is required, or the system suffers from lack of nourishment. The matter may seem a small one, but it is not so. Just as a man may go on for years with defective teeth, imperfectly masticating his food, and wondering why he suffers from indigestion, so a man may habitually live under an affliction of hurried dinners, and endure the consequent loss of health, without knowing why he is not well, or how easily the cause of his illness might be remedied.

The Canal of Joseph.—How many of the engineering works of the nineteenth century will there be in existence in the year 6,000 ? Very few, we fear, and still less those that will continue in the far-off age to serve a useful purpose. Yet there is at least one great undertaking conceived and executed by an engineer, which, during the space of 4,000 years has never ceased its office, on which the life of a fertile province absolutely depends to-day. We refer to the Bahr Joussuf—the canal of Joseph—built, according to tradition, by the son of Jacob, and which constitutes not the least of the many blessings he conferred on Egypt during the years of his prosperous rule. This canal took its rise from the Nile at Asiut, and ran nearly parallel with it for nearly 250 miles, creeping along under the western cliffs of the Nile valley, with many a bend and winding, until at length it gained an eminence, as compared with the river bed, which enabled it to turn westward through a narrow pass, and enter a district which was otherwise shut off from the fertilizing floods on which all vegetation in Egypt depends.

The northern end stood seventeen feet above low Nile, while at the southern end it was at an equal elevation with the river. Through this cut runs a perennial stream which waters a province named the Fayoum, endowing it with fertility, and supporting a large population. In the time of the annual flood a great part of the canal is under water, and then the river's current rushes in a more direct course into the pass, carrying with it the rich silt which takes the place of fertilizer.

Practical Aids to Beauty.—An excellent cosmetic for pimples is composed of the following harmless materials : Borax, powdered alum, flour of sulphur and powdered sugar ; equal parts of the first three and two parts of sugar to be dusted over the skin. Salts of tartar is excellent for cleansing the hair. One recipe is to one ounce of salt of tartar add one quart of water ; put a tablespoonful of the solution in a quart of water. Wash the hair and scalp thoroughly ; rub dry with a soft towel. When the hair is inclined to fall out clip the ends and bathe the head regularly with strong sage tea, containing a teaspoonful of salt to a pint of tea. Vigorous brushing should be regularly attended to. Half an hour at a time with a good bristle brush is not too much. The difierence between hair that is only combed and that which is well brushed is very preceptible. The washes or bleaches which turn dark hair golden are very injurious, causing it to break and in many instances become prematurely gray. The most harmless wash is Bicarbonate of Soda, and it should not be used too strong or too often. *The Woman's News.*

